# Sleep and body mass index in adolescence: results from a large population-based study of Norwegian adolescents aged 16 to 19 years

**DOI:** 10.1186/1471-2431-14-204

**Published:** 2014-08-15

**Authors:** Børge Sivertsen, Ståle Pallesen, Liv Sand, Mari Hysing

**Affiliations:** 1 Division of Mental Health, Norwegian Institute of Public Health, Kalfarveien 31, Bergen 5018, Norway; 2 Uni Health, Uni Research Bergen, P.O. Box 7810, Bergen N-5020, Norway; 3 Department of Psychiatry, Helse Fonna HF, P.O. Box 2170, Haugesund N-5504, Norway; 4 Department of Psychosocial Science, University of Bergen, P.O. Box 7807, Bergen N-5020, Norway; 5 Norwegian Competence Center for Sleep Disorders, Jonas Lies vei 65, Bergen 5021, Norway; 6 The Regional Centre for Child and Youth Mental Health and Child Welfare, Uni Health, Uni Research Bergen, P.O. Box 7810, Bergen N-5020, Norway

**Keywords:** Body mass index, Obesity, Overweight, Underweight, Sleep, Sleep duration, Insomnia, Adolescence, Epidemiology

## Abstract

**Background:**

The aim of this study was to examine the association between body mass index (BMI) and sleep duration, insomnia and symptoms of obstructive sleep apnea (OSA) in adolescents.

**Methods:**

Data were taken from a large population based study of 9,875 Norwegian adolescents aged 16–19. BMI was calculated from the self-reported body weight and categorized according to recommended age and gender specific cut offs for underweight, overweight and obesity. Detailed sleep parameters (sleep duration, insomnia, and OSA symptoms) were reported separately for weekdays and weekends. Data were analyzed using Pearson’s chi-squared test and ANOVAs for simple categorical and continuous comparisons, and multinomial logistic regressions for analyses adjusting for known confounders.

**Results:**

There was evidence for a curvilinear relationship between BMI and both sleep duration and insomnia for girls, whereas the relationship was linear for boys. Compared to the average weekday sleep duration among adolescents in the normal weight range (6 hrs 29 min), both underweight (5 hrs 48 min), overweight (6 hrs 13 min) and obese (5 hrs 57 min) adolescents had shorter sleep duration. OSA symptoms were linearly associated with BMI. Controlling for demographical factors as well as physical activity did not attenuate the associations. Additional adjustment for depression reduced the association between insomnia and obesity to a non-significant level. The evidence for a link between both underweight and overweight/obesity, and short sleep duration and OSA symptoms remained in the fully adjusted analyses. The associations were generally stronger for girls.

**Conclusions:**

This is one of the first population-based studies to investigate the relationship between sleep and BMI in adolescents while simultaneously controlling for important confounding factors. These findings require further research to investigate the temporal association between weights and sleep problems.

## Background

Both sleep problems and obesity in adolescence are growing public health concerns. The prevalence of obesity among adolescents in the US population has increased more than 3-fold over the past four decades (from 5% to 18%) [1,2]. Parallel to this epidemic of obesity, which has enormous health and economic consequences [3], there has been a similar decrease in the amount of time spent sleeping. US surveys have shown a decline in self-reported sleep duration over the past 50 years by 1.5 to 2 hours [4], and a similar decrease has been observed among adolescents [5], although the findings in children are mixed [6]. The prevalence of insomnia symptoms has shown a parallel increase in Norwegian adolescents over the last two decades [7].

Several studies have investigated the association between sleep and obesity across different age cohorts, primarily with sleep duration as the variable of interest. In a meta-analysis from 2008 covering 11 studies on children aged 2–20 years, seven of 11 studies reported a significant association between short sleep duration and obesity [8]. Four longitudinal studies have also examined this link, but the results were inconsistent regarding short sleep duration as an independent risk factor for later obesity among adolescents [9-12]. In a more recent meta-analysis [13], reviewing 15 studies on adolescents (10–19 years) investigating the effect of short sleep duration on overweight and obesity, it was concluded that the current evidence was inconclusive as to whether sleep duration was related to adolescent overweight, mainly due to methodological concerns. In addition to the link between obesity and sleep duration, there is ample evidence for obesity as a risk factor for sleep disordered breathing in clinical samples [14].

So far, insomnia (difficulty initiating and maintaining sleep) has received very little attention in relation to body mass index (BMI) in the literature, but has been associated with BMI in young females [15]. Extending on these methodological limitations, the authors of the aforementioned meta-analysis [13] emphasized the following recommendations for future research; 1) to use multiple, detailed and validated measures of sleep; 2) investigate if gender interacts with sleep duration and obesity; 3) adjust for the confounding effects of depression and physical activity; 4) provide separate analyses for both weekday and weekends; and 5) use a prospective design.

Against this background, addressing first four of the five recommendations by Guidolin and Gradisar [13], the aims of the current study were: 1) to examine the relationship between multiple and detailed sleep parameters (including sleep duration, insomnia and symptoms of obstructive sleep apnea (OSA)) and body mass index (BMI) in a large population-based sample of 16 to 19-year-old adolescents; 2) to investigate girls and boys separately and to examine potential gender differences in the associations between sleep and BMI; 3) to adjust for potential confounding factors, including physical activity and depression; and 4) to provide separate analyses for weekdays and weekends, due to the large observed differences in sleep duration on weekend nights versus school nights [16].

## Methods

In this population-based study, we used data from the youth@hordaland survey of adolescents in the county of Hordaland in Western Norway. The youth@hordaland survey is the fourth wave of the Bergen Child study, where children born 1993–1995 are followed from elementary to upper secondary school age. All adolescents and students attending secondary education during spring 2012 were invited to participate. The main aim of the survey was to assess the prevalence of mental health problems and service use in adolescents. Data were collected during spring 2012. Adolescents in upper secondary education received information via their official school e-mail address, and one classroom school hour was allocated for them to complete the questionnaire. The questionnaire was web-based and covered a broad range of mental health issues, daily life functioning, use of health care and social services, demographics, as well as a request for permission to obtain school data, and to link the information with national health registries. Uni Health collaborated with Hordaland County Council to conduct the study. The study was approved by The Regional Committee for Medical and Health Research Ethics in Western Norway. After complete description of the study to the subjects, written informed consent was obtained. All phases of study adhered to the Declaration of Helsinki.

### Sample

Of the 19,430 invited to take part, 10,200 agreed yielding a participation rate of 53%. All sleep variables were manually checked for validity and data from subjects providing obvious invalid responses were omitted for further analyses. Invalid responses included 1) sleep onset latency (SOL) + wake after sleep onset (WASO) > time in bed (TIB), and 2) negative values of sleep duration and sleep efficiency. This resulted in data from 374 subjects being omitted.

Based on previous research from the former waves of the Bergen Child Study (the same population as the current study), non-participants have been shown to have more psychological problems than participants [17], and it is therefore likely that the prevalence of mental health problems may be underestimated in the current study.

### Instruments

#### Demographic information

Gender and date of birth was identified through personal identity number in the Norwegian National Population Register. Exact age was estimated by calculating the interval of time between date of birth and date of participation. All participants indicated their vocational status, with response options being “high school student”, “vocational training” or “not in school”. Maternal and paternal education were reported separately with three response options; “primary school”, “secondary school”, “college or university”. Perceived family economy (i.e., how well off they perceive their family to be) was assessed by asking the adolescents how their family economy is compared to most others. Response alternatives were 1) “better economy”, 2) “approximately like most others”, and 3) “poorer economy”. Immigrant status was defined as having both parents born outside of Norway. Parent country of origin was indicated by the adolescent on a scroll down menu.

#### Body mass index (BMI)

BMI was calculated based on self-reported body weight (kg) divided by squared height (m^2^). The BMI was then split into 4 categories: underweight, normal weight, overweight and obesity, based on recommended age and gender specific cut-offs: ISO-BMI [18,19].

### Sleep variables

#### Sleep duration

Self-reported bedtime and rise time were indicated in hours and minutes using a scroll down menu with five minutes intervals and were reported separately for weekends and weekdays. Time in bed (TIB) was calculated by subtracting bedtime from rise time. Sleep onset latency (SOL) and wake after sleep onset (WASO) were indicated in hours and minutes using a scroll down menu with five minutes intervals, and sleep duration was defined as TIB minus (SOL + WASO). For purposes of the present study sleep duration was used both continuously and categorically: “short sleepers” (<1 SD [more than 1 SD below the mean]: 5 hours), “normal sleepers” (5 hours – 8 hours), and “long sleepers” (>1 SD [more than 1 SD over the mean]: 8 hours). Adolescents with both a weekday and weekend sleep duration of <5 hours were categorized as the “non-compensated group,” whereas children with a weekday sleep duration of <5 hours but a weekend sleep duration of >5 hours were classified as the “compensated group”, signifying adolescents using the weekends to catch up on their sleep.

#### Insomnia

Difficulties initiating and maintaining sleep (DIMS) were rated on a three point Likert-scale with the response options “not true”, “somewhat true” and “certainly true”. Given a positive response (“somewhat true” or “certainly true”), participants were then asked how many days per week they experienced problems either initiating or maintaining sleep. Duration of DIMS was rated in weeks (up to three weeks) months (up to 12 months) and a last category over a year. A joint question on tiredness/sleepiness was rated on a three point Likert-scale with response options “not true”, “somewhat true” and “certainly true”. If confirmed (“somewhat true” or “certainly true”) participants reported the number of days per week they experienced sleepiness and tiredness, respectively. Insomnia was defined according to Lichstein et al.’s Quantitative Criteria for Insomnia [20]: self-reported DIMS at least three times a week, with a duration of six months or more, in addition to reporting SOL and/or WASO of more than 30 minutes, as well as tiredness or sleepiness at least three days per week.

#### Obstructive sleep apnea (OSA) symptoms

Symptoms of OSA were estimated using two self-reported items. In addition to the requirement of reporting “true” or “partly true” on the item “I snore (or someone else says I snore)”, adolescents were defined as having OSA if they also reported “sleepiness” at least three days per week. A similar operationalization has previously been successfully applied in epidemiological studies [21,22].

### Confounders

Depression was assessed using the short version of the Mood and Feelings Questionnaire (SMFQ) [23]. The SMFQ comprises 13 items assessing depressive symptoms rated on a 3-point Likert scale. The wordings of the response categories in the Norwegian translation equals the original categories of “not true”, “sometimes true” and “true”. High internal consistency between the items and a strong uni-dimensionality have been shown in population-based studies [24], and was recently confirmed in a study based on the same sample as included in the present study [25]. The Cronbach’s alpha of the SMFQ in the current study was 0.91.

Physical activity was assessed using one item, derived from the Norwegian part of the study «Health Behaviour in School-aged Children. A WHO cross-national study (HBSC)» [26]: “During the last 7 days, how many days have you been physical active (minimum 30 minutes)?” with response ranging from “0” to “7” days. This item has previously been demonstrated to have acceptable reliability, and validity when assessed with a standardized test of aerobic fitness (the Multistage Fitness Test) [27].

### Statistics

IBM SPSS Statistics 22 for Mac (SPSS Inc., Chicago, Ill) was used for all analyses. Pearson's chi-squared test and one-way analyses of variance (ANOVA) with Bonferroni post hoc tests were used to examine differences in demographical, clinical and sleep variables between the four BMI categories “underweight”, “normal weight”, “overweight” and “obese”. Both linear and quadratic terms (weighted) were entered in the ANOVAs to show potential linear and/or U-shaped associations. Multinomial logistic regression analyses were conducted to examine the predictive effect of the sleep variables (independent variables) on BMI-categorization (dependent variable), using “normal range” as the reference category. Both crude and fully adjusted models were examined, the latter adjusting for the following covariates entered in one block: age, gender, parental education and family income, physical activity and depressive symptoms (SMFQ total score). Logistic regressions were also used to examine whether adolescents in the non-compensated group had a greater risk of overweight/obesity than the compensated group. All analyses were conducted on the whole sample as well as stratified by gender.

## Results

### Demographical and clinical characteristics of the sample

In all, 9,396 persons provided valid responses on the relevant questionnaire on sleep items and BMI. The mean age was 17.4 years, and the sample included more girls (53.3%) than boys (46.7%). The majority (98%) were high school students. 5.3% were defined as immigrants as they had both parents born outside Norway.

As detailed in Table 1, more boys than girls were categorized as overweight and obese compared to girls, while more girls were categorized as underweight (*p* < .001). Being overweight or obese was also significantly associated with lower parental education, poor family economy, as well being less physical active and higher levels of depressive symptoms (all *p*s < .001).

**Table 1:** Demographic and clinical characteristic in adolescents stratified by different categories of body-mass index (BMI)

	**Underweight**	**Normal weight**	**Overweight**	**Obesity**
N (%)	145 (1.5%)	7499 (79.8%)	1411 (15.0%)	341 (3.6%)
Age, mean (SD)	17.4 (0.8)	17.4 (0.8)^*^	17.5 (0.8)	17.5 (0.9)
BMI, mean (SD)	16.0 (0.6)	21.0 (1.9)^*^	26.5 (1.4)	32.8 (3.2)
Gender				
Girls, % (n)	8.3% (421)	77.1% (3915)^*^	11.6% (590)	3.0% (151)
Boys, % (n)	5.0% (216)	76.7% (3348)	14.7% (642)	3.6% (157)
Fathers with college education, % (n)	28.3% (41)	38.9% (2914)^*^	31.7% (447)	25.8% (88)
Mothers with college education, % (n)	23.4 (34)	34.3% (2573)^*^	25.8% (364)	16.4% (56)
Poorer family economy, % (n)	12.7% (27)	6.4% (470)^*^	8.4% (116)	15.1% (51)
Physical activity (days/wk), %				
None	25.5% (36)	42.4% (3053)^*^	35.3% (478)	26.5% (87)
1-3 days	53.9 (76)	48.8% (3511)	51.8% (701)	58.5 (192)
4 + days	20.6% (29)	8.7% (628)	12.9% /(175)	14.9% (49)
Depression (SMFQ score), mean (SD)	8.4 (6.4)	5.5 (5.5)^*^	6.4 (6.0)	8.1 (7.2)

*p < .001; p-values are based on Chi-square tests (proportions) and ANOVAS (means).

### BMI and sleep

**Figure 1:**
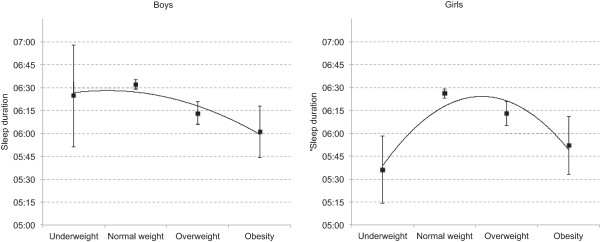
**Sleep duration stratified by BMI category in boys and girls in the youth@hordaland.** Error bars represent 95% confidence intervals. The curve shows the polynomial/curvilinear trendline (order 2).

As detailed in Table 2, there was a u-shaped association between BMI category and most sleep parameters. The average sleep duration during weekdays among obese, overweight, and underweight adolescents was 5 hrs 57 min, 6 hrs 13 min, and 5 hrs 48 min, respectively, compared to 6 hrs 29 min among adolescents with normal weight. Testing a quadratic term in the trend analysis provided strong evidence to reject a purely linear association between categorical BMI and sleep duration (*p* < .001). A similar but less pronounced u-shaped trend was observed for sleep duration during weekends (*p* = .023). Figure 1 shows the association between weekday sleep duration and categorical BMI stratified by gender: whereas a U-shaped association was found for girls (*p* < .001), the association for boys was linear (*p* < .001), not quadratic (*p* = .650). However, the interaction between gender and sleep duration for (continuous) BMI was not significant (p = 0.102).

**Table 2:** Sleep characteristic in adolescents stratified by different categories of body-mass index (BMI)

	**Underweight**	**Normal weight**	**Overweight**	**Obesity**
Sleep duration category (weekdays)				
Short sleeper (<1SD: 5 hours), % (n)	27.3% (39)	13.2%(972)^*^	18.3% (2253)	24.2% (81)
Normal Sleeper, (5–8 hours) % (n)	65.7% (94)	76.4% (5636)	72.1% (997)	66.6% (223)
Long sleeper (1 > SD: 8 hours), % (n)	7.0% (10)	10.4% (769)	9.6% (133)	9.3% (31)
Sleep duration weekdays, mean (SD)	5:48 (1:51)	6:29 (1:36)^*^	6:13 (1:43)	5:57 (1:57)
Sleep duration weekends, mean (SD)	8:28 (2:09)	8:41 (1:47)^*^	8:26 (1:59)	8:09 (2:08)
Insomnia	19.3% (28)	12.6% (943)^*^	16.4% (230)	20.1% (68)
OSA symptoms, % (n)	1.6% (2)	3.2% (194)^*^	6.8% (77)	12.8% (35)

*p < .001; p-values are based on Chi-square tests (proportions) and ANOVAS (means).

**Figure 2:**
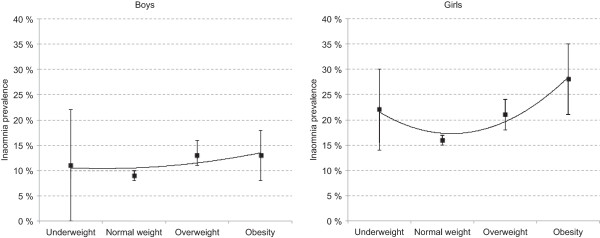
**Insomnia prevalence stratified by BMI category in boys and girls in the youth@hordaland.** Error bars represent 95% confidence intervals. The curve shows the polynomial/curvilinear trendline (order 2).

A curvilinear association was also observed for the relationship between insomnia and BMI. As detailed in Table 1, the prevalence of insomnia was higher among underweight (19.3%), overweight (16.4%) and obese (20.1%) adolescents, compared to the their normal weight peers (12.6%). As depicted in Figure 2, the curvilinear association between insomnia and BMI was present for girls (*p* = .005), not boys (*p* = .751).

BMI was linearly associated with the prevalence of OSA symptoms. In all, 12.9% and 6.8% of obese and overweight adolescents were categorized with OSA symptoms, respectively, compared to 3.5% and 1.6% among adolescents in the normal weight and underweight groups, respectively.

### Multinominal regression analyses

The results from the series of logistic regression analyses investigating the association between different sleep variables and BMI-categories are presented in Table 3. Upholding the findings from Table 2, the crude analyses showed that both short sleep duration, insomnia and OSA symptoms increased the odds of being categorized as underweight, overweight and obese, respectively

**Table 3:** **Multinomial regression analyses of sleep variables associated with BMI categories**^**§**^

	**Short sleep weekdays**^**#**^		**Short sleep weekends**^**#**^		**Insomnia**		**OSA symptoms**	
	**OR**	**95% CI**	**OR**	**95% CI**	**OR**	**95% CI**	**OR**	**95% CI**
Unadjusted analyses								
Obesity	**2.11**	1.62-2.74	**1.69**	1.10-2.60	**1.73**	1.32-2.28	**4.39**	3.00-6.44
Overweight	**1.47**	1.26-1.72	**1.31**	1.00-1.71	**1.36**	1.16-1.59	**2.18**	1.66-2.86
Underweight	**2.41**	1.65-3.52	0.88	0.37-2.06	**1.65**	1.09-2.51	0.49	0.12-2.02
Adj. for demographics (age, gender, parental education and family income)								
Obesity	**2.11**	1.61-2.75	**1.64**	1.06-2.55	**1.75**	1.32-2.31	**4.28**	2.91-6.29
Overweight	**1.51**	1.29-1.77	**1.33**	1.02-1.75	**1.46**	1.24-1.71	**2.10**	1.59-2.77
Underweight	**2.22**	1.51-3.27	0.84	0.36-1.99	1.49	0.97-2.27	0.53	0.13-2.15
Adj. for demographics + physical activity								
Obesity	**2.04**	1.56-2.68	**1.79**	1.15-2.78	**1.73**	1.30-2.30	**4.25**	2.86-6.30
Overweight	**1.49**	1.27-1.75	1.31	0.99-1.73	**1.44**	1.22-1.69	**2.10**	1.58-2.79
Underweight	**2.12**	1.43-3.14	0.89	0.37-2.10	1.34	0.97-2.09	0.26	0.04-1.87
Adj. for demographics + depression								
Obesity	**1.60**	1.21-2.12	1.37	0.88-2.14	1.25	0.93-1.69	**3.53**	2.37-5.25
Overweight	**1.34**	1.14-1.58	1.21	0.91-1.59	**1.26**	1.07-1.50	**1.96**	1.48-2.60
Underweight	**1.87**	1.25-2.81	0.72	0.30-1.70	1.12	0.71-1.75	0.46	0.1-13.88
Adj. for demographics, physical activity and depression								
Obesity	**1.58**	1.19-2.09	1.45	0.92-2.28	1.25	0.93-1.69	**3.69**	2.47-5.51
Overweight	**1.33**	1.12-1.57	1.20	0.90-1.59	1.24	1.05-1.47	**1.98**	1.49-2.64
Underweight	**1.78**	1.18-2.68	0.75	0.32-1.80	1.06	0.67-1.67	0.23	0.03-1.69
Adj. for demographics, physical activity, depression and two other sleep variables (sleep duration, insomnia or OSA symptoms)^$^								
Obesity	**1.51**	1.10-2.08	1.42	0.87-2.31	1.14	0.83-1.58	**3.52**	2.35-5.26
Overweight	**1.23**	1.02-1.47	1.16	0.85-1.58	1.20	0.99-1.44	**1.90**	1.42-2.53
Underweight	**1.88**	1.20-2.95	0.91	0.38-2.21	0.98	0.60-1.59	0.22	0.03-1.60

§Reference: Normal weight range.

^#^Compared to normal sleep duration (>5 hours to < 8 hours).

^$^For short sleep, the effect was adjusted additionally for insomnia and OSA symptoms; for insomnia, the effect was additionally adjusted for sleep duration and OSA etc.

Bold font denotes statistical significant OR at p < .05.

Adjusting for confounders, including socio-demographics, physical activity and depressive symptoms, reduced several of the odds-ratios. Depression was the one confounder that explained most of the reductions in ORs; socio-demographical factors and physical activity did not, or only slightly, attenuate the associations. However, the association between weekday short sleep duration and BMI categorization remained significant also in the fully adjusted analyses (ORs ranging from 1.23 to 1.88; see Table 3 for details), as was the case for OSA symptoms and obesity (OR = 3.52), and OSA and overweight (OR = 1.90). The association between BMI categorization, and insomnia and short sleep duration on weekends did not remain significant in the fully adjusted analyses.

We also examined the association between sleep compensation during weekends and risk of obesity/overweight. Using the compensated group of adolescents as a reference, the crude OR for obesity was 1.44 (95% CI: 0.88– 2.35) for those with persistently short sleep duration (<5 hours) during weekdays and weekends (non-compensated group). The corresponding ORs for overweight and underweight were OR = 1.10 (95% CI 1.80-1.51) and OR = 0.55 (95% CI 0.23-1.33), respectively.

### Gender differences in the association between obesity and sleep

**Figure 3:**
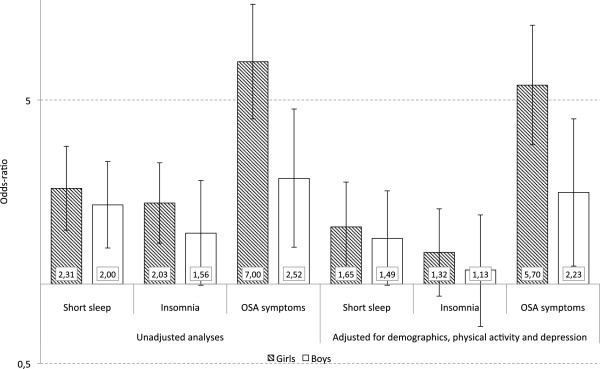
**Gender differences in the association between sleep variables and obesity.** Bars represent odds-ratios from the gender-stratified multinomial regression analyses (outcome: obesity compared to normal weight) and error bars represent 95% confidence intervals (Y-axis has a logarithmic scale).

The associations between OSA symptoms and obesity were stronger among girls (crude OR = 7.00, 95% CI: 4.24-11.56) compared to boys (crude OR = 2.52, 95% CI: 1.38-4.62) (see Figure 3). These differences were present also in the adjusted analyses. As evident from Figure 3, although a similar trend of gender differences were observed for the associations between obesity, and insomnia or short sleep duration, these were not statistically significant.

## Discussion

Using data from a large population-based study of Norwegian adolescents aged 16–19 years, we found a strong U-shaped association between BMI and both sleep duration and insomnia for girls, whereas the relationships were linear for boys. Most effects remained significant after adjusting for important confounders, including socio-demographic variables, depression and physical activity, although insomnia was no longer related to BMI after adjusting for depression. All associations between BMI and sleep were in general stronger for girls, especially for OSA symptoms.

The finding of a U-shaped association between sleep duration and BMI has to the best of our knowledge not been previously demonstrated in adolescents, although it is in accordance with the only previous study investigating this in children [28], as well as with several studies on adults [29-31]. There are several potential pathways, both biological and behavioral, that may explain how sleep problems duration may be related to overweight/obesity. In terms of biological explanations, several laboratory studies have demonstrated how sleep restriction is linked to alterations in the production of hormones that control appetite, such as leptin and ghrelin, which may lead to subsequent weight gain [32,33]. It has also been suggested that the association between short sleep and adolescent obesity is stronger at the upper tail of the BMI distribution [10], implying that adolescents with excessive weight are at risk of further increases in BMI due to reduced sleep.

Short sleep duration may also impact eating patterns, with a stronger preference for fatty food when tired. Further, being awake more gives many opportunities for snacking and late night meals that would add to the total calorie intake. Although the relative changes in eating and hormones may be minor, it has been demonstrated that even small changes in eating patterns may cumulatively alter the energy balance, thereby increasing the risk of obesity among the adolescents [34]. It has also been suggested that daytime tiredness and fatigue in addition to changed eating patterns may result in less physical exercise, which in turn reduces the body’s total energy expenditure, and thereby increasing the risk of subsequent obesity [35]. This notion is a line with a recent study that showed that poor sleep decreased engagement in exercise the next day [36]. However, as both demonstrated in the current study, as well as in a previous review [37], physical activity only slightly attenuated the association between BMI and sleep.

The association between underweight and short sleep duration is interesting in light of the current focus on overweight and obese adolescents. Sleep time breathing disorders have been reported to be as common among underweight as overweight children [38], and the risk of OSA has been found to higher for both over- and underweight children relative to normal weight children [39]. However, OSA did not mediate the relationship between underweight and short sleep duration in the current study, as the prevalence of OSA was very low in the underweight group (1.6% compared to 3.2% in the normal weight group).

While the pathways and mechanisms between sleep and overweight and obesity are well described, less is known of the pathways between sleep and underweight. Still there has been suggested that a low calorie intake may be linked with low levels of sleep inducing gut-peptids such as cholecystokinin as well as increase in wake agents such as orexin [40]. Previous research has found depression to increase the risk of both obesity [41] and sleep problems [42], making it important to include depression as an adjustment variable when attempting to investigate a potential link between sleep and obesity. Indeed, the current study found depression to be the single most important factor in explaining the observed association, but showed differential associations according to sleep problems. While the insomnia-BMI association was reduced to a non-significant level when adjusting for depression, the associations with OSA symptoms and sleep duration were only slightly attenuated. This close and complex connection between insomnia and depression is supported by mounting data pointing to a reciprocal relationship between the two conditions, as demonstrated both in adolescents and adults [42-45]. The current finding is also line with previous studies investigating how depression may explain the link between short sleep duration and obesity [46]. However, longitudinal studies are needed to cannot adequately explain the complex relationship among insomnia, depression and BMI.

While the majority of the previous studies have limited the assessment of sleep to its duration, the present study included a broader selection of sleep variables. The strength of the relation between sleep measures, and the role of confounding factors varied across type of sleep problems. Overweight and obese children had the highest odds of OSA symptoms. This is in line with previous clinical studies that find a strong association between OSA and overweight in adolescence [47]. The increased odds were still high with a more than threefold increase in odds, even after adjusting for demographic variables, physical activity and depression. Although the causal direction cannot be explored in the current study, previous studies have found obesity to be a risk factor for sleep disordered breathing, possible due to pharyngeal lymphoid tissue enlargement [48].

The prevalence of the various sleep problems differed by gender. Girls had a higher rate of insomnia, while males have on average reported shorter sleep duration. The literature has been inconsistent regarding gender specific relations between sleep and obesity, with some studies finding stronger associations among males. In one of the few previous studies investigating the relationship between BMI and sleep in adolescents and young adults, there were gender specific patterns between type of sleep problems, with men showing an association between BMI and sleep duration, while women’s BMI were related to problems initiating and maintaining sleep [15]. In contrast, the current study found that the associations were stronger for girls across all sleep measures, but especially so for OSA symptoms. Although there was a tendency for the same gender pattern also being present for insomnia and short sleep, these differences were not statistically significant. Still, these findings suggest that further investigations with regards to gender-specific mechanisms and pathways are warranted. For instance, it has been suggest that gender plays a role in how sleep duration specifically affects body composition. According to Skidmoe and colleagues [49], insufficient sleep among adolescent boys influences fat body mass more than lean mass. Thus, assessing weight changes solely by BMI for could mask the relationship between sleep duration and adiposity for males. The authors recommend using multiple body composition measures including Fat Body Mass (FBM) in order to adjust for gender interactions.

There are some methodological limitations of the present study that deserves mention. First, height and weight were based on self-report. Although physical measurements would be preferable, a recent study of adolescents showed that self-reported height and weight are indeed a suitable proxy to estimate the prevalence of overweight and obesity [50]. However, it remains unknown whether self-reporting height and weight was influenced by bodyweight status in the present study. Studies on students show that females and subjects with high BMI tend to underreport weight relatively to their counterparts [51] although studies on children has shown them to be quite accurate in the self-report of height and weight [52]. Second, depression was assessed by a self-report instrument, the SMFQ, thus the lack of clinical interview in confirming a clinical diagnosis of depression is a limitation of the present study. In relation to this, the absence of sleep items in the SMFQ is both a limitation and an asset for the purpose of this study. A conventional depression rating scale, including sleep problems as a symptom, would by definition represent circularity, and make the interpretation of the results more ambiguous. Tiredness was included in the SMFQ, but the association to several sleep parameters was not higher for this item than for other depressive symptoms. Third, while an association between BMI and sleep was demonstrated, conclusions regarding the temporal sequence warrant longitudinal studies with multiple measurements. Although most of the literature has investigated whether sleep (exposure) has an effect on BMI (outcome), it is also possible that the reverse directionality may hold. Therefore, more prospective studies are needed to provide clearer insights into causality (i.e., do sleep changes predict weight changes, or vice versa). Fourth, while the definition of insomnia was based on published quantitative criteria, it was not based on a structured interview, which of course is difficult to employ in a population-based study. Future research is needed to establish if the reported patterns hold among other ethnic groups. The use of both SOL and WASO to estimate exact sleep duration was a significant strength of the current study, as most population based studies on sleep rarely provide such detailed measures. Although self-reported sleep parameters, including SOL and WASO typically differ from those obtained from objective assessments [53], recent studies have showed that such self-report sleep assessments can be recommended for the characterization of sleep parameters in both clinical and population-based research [54]. Also, the accuracy of self-reported SOL and WASO are generally better among adolescents than in older adults [55], and a study of young adolescents in Hong Kong recently found good agreement between actigraphy measured and questionnaire reported sleep durations [56]. In addition to being used continuously, sleep duration was also classified into 3 categories based on statistical distribution (standard deviations), and not according to norms or recommendations. It should be mentioned that the latter approach might imply a risk of e.g. those being classified with “normal sleep” as still being sleep deprived.

The use of the Quantitaive Research Criteria for Insomnia [20] is also a major strength of the study, not limiting sleep problems to self-reported single items of initiating and maintaining sleep as has been used in previous studies [15]. It should also be noted that all data in the present study were based on self-reports, which renders the results susceptible to influence from the common method bias [57]. Also, attrition from the study could affect generalizability, with a response rate of about 53% and with adolescents in schools overrepresented. The problem with non-participation in survey research seems unfortunately to be on the rise [58]. Official data show that in 2012, 92% of all adolescents in Norway aged 16–18 attended high school [59], compared to 98% in the current study. Based on previous research from the former waves of the Bergen Child Study (the same population as the current study), non-participants have also been shown to have more psychological problems than participants [17], and it is therefore likely that the prevalence of mental health problems may be underestimated in the current study. We did not have any information with regards to representatives beyond mental health comparisons and school attendance. As such, the findings of the current study might not generalize to adolescents not in school, or to those with substantial psychological problems. Finally, the cross-sectional design of the study restricts causal attributions, and prospective studies are still needed to disentangle the temporal relationship.

The co-occurrence of sleep and obesity, both major public health problems indicate that a broad assessment could be indicated in adolescents presenting with these problems. Future studies could assess if altering sleep or obesity has an impact on the other, and this could also shed light on the mechanisms and temporal relationships.

## Conclusion

In conclusion, this is the first population-based study to investigate the relationship between a range of sleep parameters and BMI in adolescents. Although many of the observed associations were reduced to a non-significant level when adjusting for depression, both short sleep duration and nocturnal wake time remained independent risk factors for both obesity and underweight among adolescent boys and girls. Further research to investigate the temporal association between overweight and sleep problems is warranated.

## Competing interests

The authors declare that they have no competing interests.

## Authors’ contributions

Author BS and MH were involved in acquisition of data. Authors BS and MH were responsible for conception and design of the study, conducted the statistical analysis and drafted the manuscript. Authors SP an LS gave critical revision of the manuscript for important intellectual content. Authors BS and MH had full access to all the data in the study and takes responsibility for the integrity of the data and the accuracy of the data analysis. All authors read and approved the final manuscript.

## Pre-publication history

The pre-publication history for this paper can be accessed here:

http://www.biomedcentral.com/1471-2431/14/204/prepub
